# High report of miscarriage among women living with HIV who want to conceive in Uganda

**DOI:** 10.1186/s13104-018-3857-9

**Published:** 2018-10-22

**Authors:** Sarah Finocchario-Kessler, Kathy Goggin, Vince Staggs, Rhoda K. Wanyenze, Jolly Beyeza-Kashesya, Deborah Mindry, Josephine Birungi, Glenn J. Wagner

**Affiliations:** 1 Department of Family Medicine, University of Kansas Medical Center, Kansas City, USA; 2 Health Services and Outcomes Research, Children’s Mercy Kansas City, Kansas City, USA; 3 Schools of Medicine and Pharmacy, University of Missouri–Kansas City, Kansas City, USA; 4 Department of Disease Control and Environmental Health, Makerere University School of Public Health, Kampala, Uganda; 5 Mulago Hospital Department of Obstetrics and Gynaecology, Makerere University College of Health Sciences, Kampala, Uganda; 6 Los Angeles Center for Culture and Health, University of California, Los Angeles, USA; 7 The AIDS Support Organization, Kampala, Uganda; 8 RAND Corporation, Santa Monica, USA

**Keywords:** Miscarriage, Early miscarriage, HIV, Pregnancy outcomes, Serodiscordant, Uganda

## Abstract

**Objective:**

Data on early miscarriage incidence is limited due to various social and methodological barriers. We report on 24-month pregnancy outcomes of 299 female Ugandan HIV clients in committed relationships with an intention to conceive. Miscarriage data are reported as auxiliary findings to a larger study (5R01HD072633).

**Results:**

127 (42%) participants reported a pregnancy during the study; among the remaining 172, 82 indicated they stopped trying to conceive, and 16 dropped out prior to month 24. Of the 127 pregnancies, 55 (43%) resulted in live births, 67 (53%) in spontaneous miscarriage, 1 (< 1%) in stillbirth, 1 (< 1%) in abortion, and 3 (2%) in unknown outcomes. Three-quarters (75%) of miscarriages for which time until miscarriage was available were reported to occur in the first trimester (mean = 11.3 weeks gestation). The 67 participants who reported a miscarriage tended to be older (mean 33 vs. 30 years), but the significance of age did not persist after adjusting for multiple tests. We observed relatively low rates of pregnancy and high rates of miscarriage among this cohort of HIV-positive women wanting to conceive. Rigorously designed studies are needed to better understand the observed high rate of early miscarriage among HIV-infected women.

## Introduction

In Uganda, one-third of women report at least one miscarriage (spontaneous or induced) or stillbirth during their lifetime [1]. Data is limited due to measurement inconsistency (mostly retrospective recall) [1–4], limited clinical documentation of early miscarriage [4, 5], stigma and legal considerations [6–8] that influence reporting of spontaneous vs. induced miscarriages, and a paucity of studies designed to prospectively measure miscarriage as a primary outcome.

This brief communication reports on 24-month pregnancy outcomes of a longitudinal cohort of HIV-positive women with an intention to conceive who were followed for 24 months. Miscarriage was not a targeted outcome of this study, thus we report these data in light of several limitations to be addressed in future studies. We report the incidence and timing of pregnancy and miscarriage among female participants, and examine predictors of miscarriage.

## Main text

### Study design and setting

Data were collected as part of a prospective cohort study, which assessed use of safer conception methods (SCM) (e.g., timed unprotected intercourse, manual self-insemination), and barriers and facilitators to SCM among people living with HIV who want to conceive. We followed 400 patients receiving HIV care at The AIDS Service Organization clinics in Kampala and Jinja, Uganda. Data for this manuscript, however; are limited to the n = 299 female participants. Eligible participants were at least 18 years old, married or in committed heterosexual relationships, and reported an intention to conceive over the following 24 months. Clients were eligible regardless of their partner’s HIV status, and provided written informed consent before participating in the baseline survey. Follow-up surveys were conducted at months 6, 12, 18 and 24. Analyses were limited to participants’ first reported pregnancy between baseline and the 24-month survey. If the client became pregnant and delivered a child, an additional interview was conducted within 1 month of the delivery. A more detailed description of the study methods and measures has been previously published [9, 10].

### Measures

During follow-up surveys, participants were asked if they became pregnant since the prior survey. Pregnancy outcomes were assessed at each follow-up: still pregnant, miscarriage (documenting spontaneous or induced, and gestational week at miscarriage), stillbirth (gestational week at stillbirth), or live birth. We examined the timing of pregnancy initiation by calculating the number of weeks between study enrollment and the date the respondent learned of her pregnancy; date of conception could not be estimated for most pregnancies due to missing data. In 10 cases for which the date the respondent learned of pregnancy was missing, we estimated this date using the midpoint of the 6-month interval preceding the survey in which the pregnancy was reported to have begun (e.g., month 3 for pregnancies that were reported to have started “since the prior survey” at the month 6 survey). Pregnancies and pregnancy outcomes were not clinically confirmed. Women reporting a miscarriage were asked to estimate the week of gestation at which the miscarriage became known.

### Analysis

**Fig. 1:**
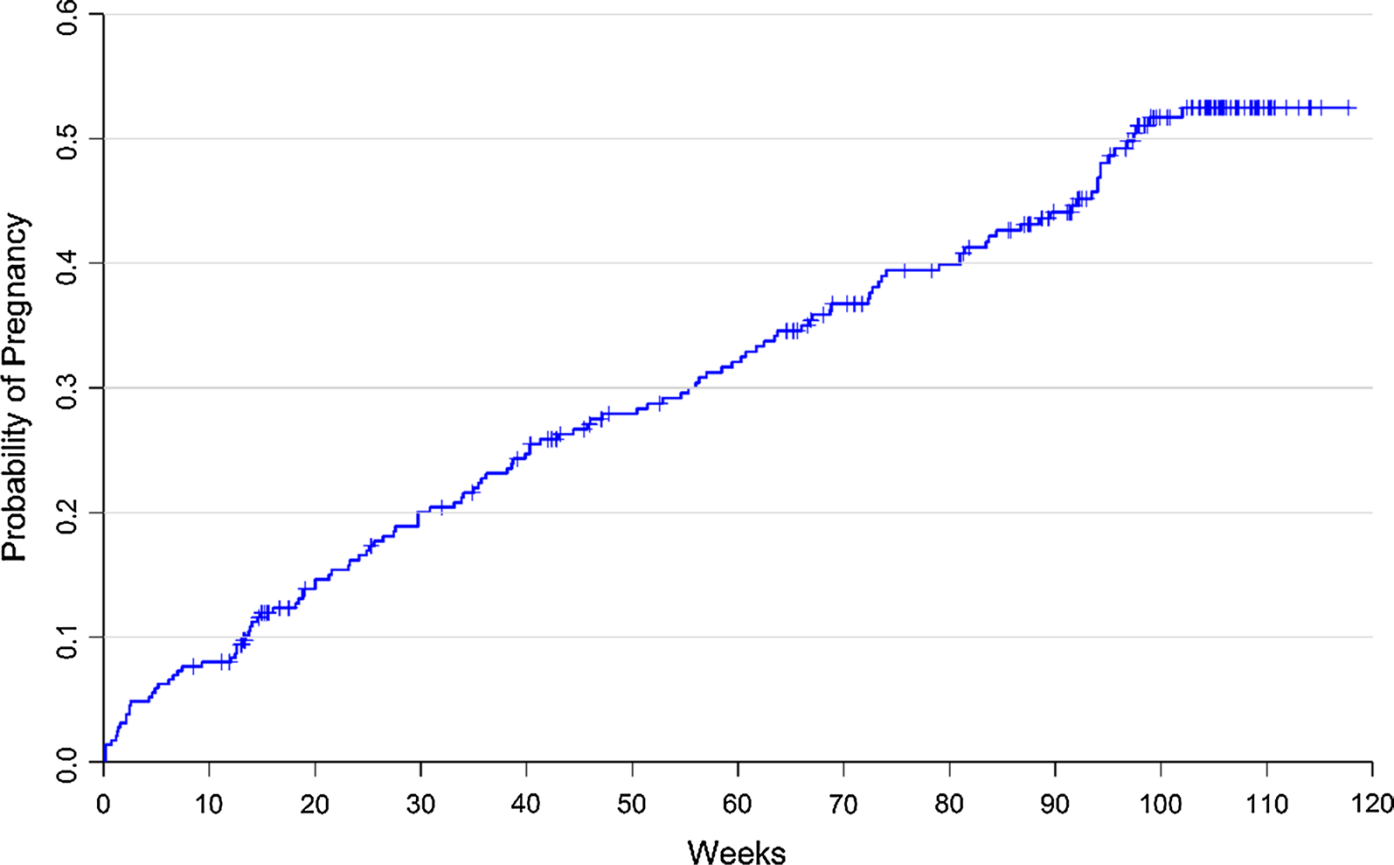
A Kaplan–Meier curve for time to first pregnancy

**Fig. 2:**
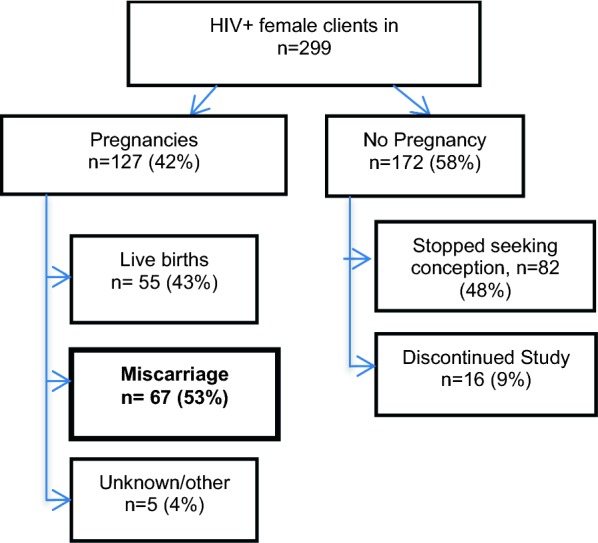
Pregnancy outcomes flow chart

**Fig. 3:**
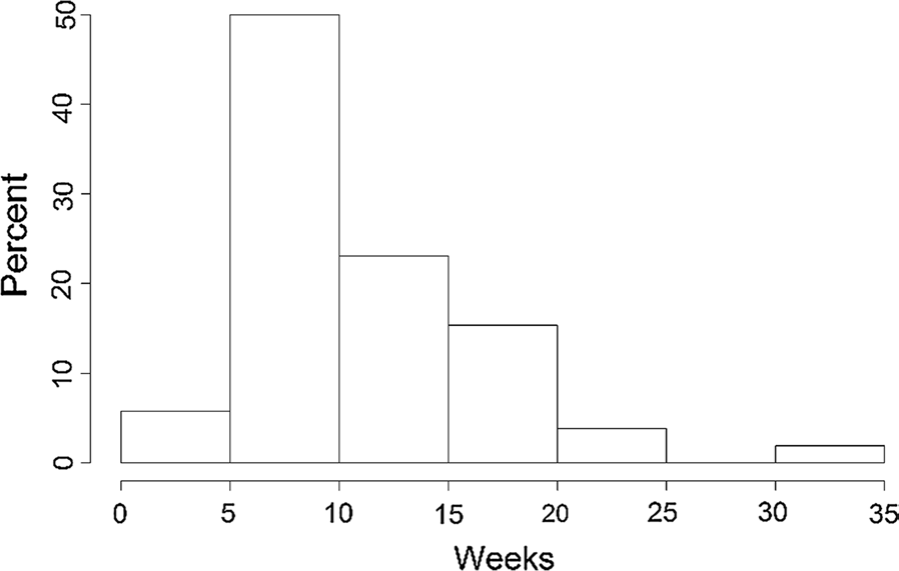
Histogram of time to miscarriage (weeks)

Sample characteristics were summarized, and a Kaplan–Meier curve was used to display time to pregnancy, with respondents who stopped trying to become pregnant censored at the time of the survey in which they reported they were no longer trying (Fig. 1). Figure 2 depicts the incidence of reported pregnancies and miscarriages, while Fig. 3 displays the reported gestational week of miscarriage to highlight the timing of these events.

Using logistic regression, we explored demographics and health characteristics (education; partner HIV status; number of past deliveries; previous miscarriage; chart-abstracted CD4 count and ART status at baseline) and psychosocial measures (social support, depression, and perceived childbearing stigma from their community) as potential predictors of miscarriage of stillbirth [10].

### Baseline characteristics

Among the n = 299 female participants, mean age was 32.0 years (SD = 6.1, range 18–48), 59% had any secondary level education, and all had at least one child (median = 2 children; mean = 2.4). Average CD4 cell count was 453 cells/mm^3^ and 59% were on ART at baseline. All were in committed relationships [38.1% married and 35.1% reported that either they (n = 3) or their male partners (n = 102) had another spouse/partner]. Three-quarters had disclosed their HIV status to their male partner. 35% reported having an HIV-positive partner, 28% had an HIV-negative partner and 37% did not know their partner’s status. At enrollment, 92% desired a child within 12 months.

### Incidence of pregnancy

By month 24, a total of 127 women had become pregnant at least once. Only first pregnancies during the course of the study were used in these analyses. Of the 172 (58%) female participants who did not report a pregnancy, 82 stopped seeking conception at some point during the study for various reasons (no longer in relationship or partner died; client and/or partner no longer desire child; declining health; unstable finances), and 16 discontinued study participation prior to month 24. Two of the women who discontinued participation and 7 who stopped trying to conceive were determined by the study obstetrician gynecologist to be infertile at some point during the study.

### Incidence of miscarriage

Of the 127 first pregnancies, 55 (43%) resulted in live birth, 67 (53%) in miscarriage, 2% Other (< 1% in stillbirth, and < 1% in abortion). Pregnancy outcome was unknown for the remaining 3 (2%) pregnancies. Rates of miscarriage were similar for pregnancies where the HIV status of the male partner was positive (36%), negative (28%), and missing or unknown (38%). Rates of miscarriage were higher among women on ART at baseline (59%) verses those who were not (43%), but the difference was not statistically significant.

### Timing of miscarriage

The timing of miscarriage (in gestational weeks) was available for 52 of the 67 miscarriages. As shown in the histogram in Fig. 3, most miscarriages occurred during the first trimester, with 19% occurring by week 6, 40% by week 8, 56% by week 10, and 75% by week 13. Among these 52 miscarriages, time to miscarriage was longer for those on ART at baseline (n = 33, median 12 weeks, IQR 8–16) than those not on ART at baseline (n = 19, median 8 weeks, IQR 6–12; Wilcoxon *p* value = 0.015). The date of first antenatal visit was unavailable for 69% of all pregnancies and for 85% of miscarriages. But among the 40 women for whom this information was available, only 18 (45%) reported antenatal care during the first trimester (median gestational age at start of ANC = 17.4 weeks). Thus, most miscarriages occurred before antenatal or prevention of mother-to-child transmission (PMTCT) care typically began; limiting the ability to evaluate length of ART exposure prior to miscarriage.

### Predictors of miscarriage

The 67 female participants who reported a miscarriage were older on average at baseline (33 vs. 30 years) compared to female participants who did not miscarry. Education, partner HIV status, number of past deliveries, previous miscarriage, CD4 count, ART, social support, perceived community stigma regarding childbearing, and depression were also assessed in unadjusted and adjusted logistic regression analyses, but only maternal age was significantly associated with miscarriage (AOR 1.09, 95% CI (1.01, 1.17)). The statistical significance of age did not persist after adjusting for multiple testing.

This cohort of women living with HIV who wanted to conceive achieved relatively low rates of pregnancy, and high rates of miscarriage among those who became pregnancy. After 2 years of prospective follow-up, less than half (42%) of women living with HIV reported a pregnancy, and 54% of pregnancies resulted in pregnancy loss (53% spontaneous miscarriage, < 1% stillbirth, < 1% abortion).

This reported miscarriage rate of 53% among reproductive aged women living with HIV is exceptionally high. Miscarriage was significantly higher in the absence of antenatal care (65% vs. 25%), but the early timing of the miscarriages (75% by 13 weeks gestation) likely pre-empted formal enrollment in antenatal care for many. We explored several potential predictors of miscarriage among HIV-infected female participants, but only age emerged as significant, and only without adjustment for multiple testing.

In the general population, an estimated 15–25% of clinically recognized pregnancies end in miscarriage [11], and this rate increases sharply for women age 35 and older [12]. The prospective follow up in this study (preconception through 24 months) may at least partially explain the higher miscarriage incidence reported in this study as it enabled documentation of early miscarriage. These early miscarriages would not typically be reported in routine clinical records since most occurred before the second trimester, when antenatal care is typically initiated in Uganda [5]. In research and surveillance efforts, miscarriages are typically assessed by self-report and often years after the event [3, 13]. Furthermore, reporting on miscarriage often combines miscarriage (without distinction between induced and spontaneous) with stillbirth, or outcomes are inconsistently reported in units that are difficult to meaningfully compare across studies [2, 14, 15].

## Limitations

There are several limitations to be considered when contextualizing these findings. As a secondary outcome, the study was not designed to rigorously measure pregnancy outcomes and relied on self-reported rather than clinically confirmed pregnancies. Data regarding the signs and symptoms that lead a woman to conclude she had miscarried were also limited. ART regimen details were not available to explore associations with miscarriage. The underreporting of induced miscarriages is possible due to stigma and legal status [16, 17]; however, induced miscarriages were expected to be low given participants expressed interest to conceive at study entry. The total number of miscarriages was relatively small for analyses and data regarding the timing of miscarriage were incomplete for the sample. Our recently initiated safer conception counseling intervention trial (NCT03167879) is designed to address these identified limitations.

Even in the context of these methodologic limitations, the high rate of miscarriage observed in this study among women living with HIV in Uganda can generate hypotheses for investigation in future studies. Recognizing the limitations of this study, we hope these preliminary analyses will lead to more rigorous evaluations of miscarriage, particularly in early pregnancy, to inform strategies to optimize pregnancy outcomes.

## Abbreviations

ART: antiretroviral therapy
HIV: human immunodeficiency virus
IQR: interquartile range
PMTCT: prevention of mother to child transmission
SCM: safer conception methods
SD: standard deviation
